# CD86 in Dendritic Cell-Mediated Cancer Immunity: From Maturation Marker to Functional Regulator

**DOI:** 10.3390/biomedicines14071588

**Published:** 2026-07-16

**Authors:** Ting-Wei Wu, Chu-Hsin Chuang, Yi-Hui Wu

**Affiliations:** 1 Division of Gastroenterology and Hepatology, Department of Internal Medicine, Chi Mei Hospital, Liouying, Tainan 73657, Taiwan; a7s8d99@gmail.com; 2 Department of Gastroenterology and General Surgery, Chi Mei Hospital, Liouying, Tainan 73657, Taiwan; 3 Medical Research Center, Chi Mei Hospital, Liouying, Tainan 73657, Taiwan; 4 Department of Obstetrics and Gynecology, National Cheng Kung University Hospital, College of Medicine, National Cheng Kung University, Tainan 70403, Taiwan; 5 Center of General Education, Min-Hwei Junior College of Health Care Management, Tainan 73658, Taiwan

**Keywords:** CD86, dendritic cells, cancer immunotherapy, tumor microenvironment, immune regulation, CTLA-4, co-stimulatory signaling, trans-endocytosis, MARCH1, immune checkpoint

## Abstract

Dendritic cell (DC)-based cancer immunotherapy remains limited by heterogeneous immune responses and variable clinical efficacy. CD86, a key co-stimulatory molecule, is traditionally regarded as a marker of dendritic cell maturation; however, accumulating evidence suggests that CD86 expression is regulated by immune checkpoint interactions, inflammatory signaling, and tumor microenvironment-associated immune modulation. In this review, we summarize current evidence regarding the molecular mechanisms governing CD86 regulation, including MARCH1-mediated ubiquitination and CTLA-4-mediated trans-endocytosis, and discuss how suppressive cytokines, hypoxia, and metabolic stress influence dendritic cell function within the tumor microenvironment (TME). We further review the heterogeneity of CD86 regulation across dendritic cell subsets and immune contexts, as well as its potential relevance in secondary lymphoid organs and tumor-associated immune responses. In addition, we discuss current evidence regarding soluble CD86 (sCD86) and its reported associations with immune activation and dysregulated immune states in cancer. Current evidence supports that CD86 regulation is shaped by integrated co-stimulatory signaling, immune checkpoint interactions, and tumor microenvironment-associated suppression. Importantly, CD86 may function not only as a dendritic cell maturation marker but also as a dynamic immunoregulatory molecule with context-dependent implications in cancer immunity. However, substantial uncertainties remain regarding its mechanistic role, prognostic value, and therapeutic relevance across different tumor settings. Future mechanistic and translational studies are needed to clarify these unresolved issues.

## 1. Introduction

Cancer immunotherapy has emerged as a major therapeutic strategy by harnessing the host immune system to recognize and eliminate malignant cells [1,2]. Among immune cell populations, dendritic cells (DCs) are important orchestrators of anti-tumor immunity [3]. As professional antigen-presenting cells, DCs bridge innate and adaptive immunity by processing tumor antigens and presenting them to naïve T cells through major histocompatibility complex (MHC) molecules, thereby initiating tumor-specific T-cell responses [4].

Based on these immunological properties, DC-based immunotherapy has been widely investigated across multiple malignancies [5]. Since its clinical introduction in the 1990s, numerous trials have evaluated DC therapy in cancer treatment [6]. However, although encouraging immunologic responses have been observed in selected studies, the overall clinical efficacy of DC-based immunotherapy remains inconsistent [7,8]. Several factors may contribute to these variable outcomes, including inadequate DC maturation [9,10,11,12], poor lymph node homing, and immunosuppressive signaling within the tumor microenvironment (TME) [1,13,14].

Effective T-cell activation requires coordinated signaling events, including antigen presentation, co-stimulatory signaling, and cytokine-mediated immune polarization [15,16,17]. Among these mechanisms, co-stimulatory molecules expressed on dendritic cells provide the second signal required for full T-cell activation. CD86, a member of the B7 family, is one of the key co-stimulatory ligands that interacts with CD28 and CTLA-4 on T cells to regulate immune activation and immune tolerance [5,16,18].

Although CD86 is widely used as a phenotypic marker of dendritic cell maturation, its precise role in shaping anti-tumor immune responses remains incompletely understood [5,19,20,21]. In this review, we summarize current evidence regarding the molecular regulation and immunological functions of CD86, with particular emphasis on tumor microenvironment-mediated immune regulation and dendritic cell-associated cancer immunity. We also discuss emerging perspectives on context-dependent CD86 regulation and their potential relevance to dendritic cell biology and tumor-associated immune regulation.

## 2. CD86 as a Co-Stimulatory Molecule in Anti-Tumor Immunity

Although the intracellular signaling pathways associated with CD86 are still being elucidated, substantial progress has been made in understanding its role in T-cell priming and immune tolerance.

The co-stimulatory axis is primarily governed by CD28 and CD152 (cytotoxic T-lymphocyte-associated protein 4, CTLA-4), which exert opposing effects on T-cell activation and inhibition, respectively [22]. Both receptors share two ligands: CD80 and CD86. Despite overlapping functional roles, CD80 and CD86 share only approximately 25% sequence identity [23,24]. CD86 is constitutively expressed on professional antigen-presenting cells (APCs), including dendritic cells, B cells, and macrophages, and is rapidly upregulated following stimulation with lipopolysaccharide (LPS) or other Toll-like receptor (TLR) ligands [25,26]. Importantly, dendritic cells are heterogeneous and can be broadly classified into conventional dendritic cells (cDC1 and cDC2), plasmacytoid dendritic cells (pDCs), and monocyte-derived dendritic cells (moDCs) [27,28,29,30]. cDC1 is specialized in cross-presentation and CD8+ T-cell activation, whereas cDC2 primarily supports CD4+ T-cell priming [31,32]. In contrast, pDCs are major producers of type I interferons, and moDCs are typically generated under inflammatory conditions [33,34,35]. These subset-specific differences indicate that CD86 expression should be interpreted in a lineage- and context-dependent manner rather than as a uniform feature across all APC populations.

Beyond lineage identity, these dendritic cell subsets also differ substantially in their CD86 expression dynamics and functional consequences in tumor immunity. cDC1 is generally associated with stronger CD86 upregulation during cross-presentation and is critical for cytotoxic CD8+ T-cell priming, particularly in tumors responsive to immune checkpoint blockade. In contrast, cDC2 often displays more heterogeneous CD86 expression and may preferentially regulate CD4+ T-cell differentiation, including Th1, Th2, or Treg-associated responses depending on local cytokine conditions. pDCs may exhibit reduced co-stimulatory capacity under chronic inflammation and can contribute to immune tolerance, whereas moDCs generated in inflammatory tumor environments may display context-dependent plasticity. These subset-specific differences suggest that CD86 regulation should be interpreted not only by expression intensity but also by dendritic cell lineage and tumor context.

Compared with CD80, CD86 is generally characterized by earlier and more abundant surface expression following immune activation, which may contribute to more rapid CD28-mediated co-stimulatory signaling during early T-cell priming [25,36,37]. This rapid induction enables effective activation of naïve T cells and supports regulatory T-cell (Treg) modulation, even in the presence of elevated CD152 expression. Consequently, CD86 is considered to play an important role in driving T helper 1 (Th1) polarization and enhancing natural killer (NK) cell activation [19,25,26,36]. CD80 forms a bivalent homodimer, whereas CD86 exists as a monovalent monomer. Owing to its bivalent configuration, CD80 exhibits markedly higher affinity and avidity for CD152 through dimer–dimer interactions (~0.2 μM), approximately tenfold stronger than the monovalent CD86–CD152 interaction (~2 μM) [37]. Under steady-state conditions, CD80 therefore preferentially engages CD152 on T cells, contributing to immune tolerance and the prevention of autoimmunity [25,38].

Under non-inflammatory conditions, DCs maintain low CD86 surface expression through immunoregulatory mechanisms [18,39]. The membrane-anchored E3 ubiquitin ligase MARCH1 ubiquitinates CD86 at lysine 267 in response to autocrine IL-10, thereby promoting its internalization and degradation [26,40,41]. Consistently, MARCH1-deficient mice exhibit markedly elevated CD86 expression. By favoring CD80–CD152 interactions, this steady-state regulatory network raises the activation threshold and prevents inappropriate immune activation [25]. Importantly, this regulatory framework may have direct implications for tumor immunology, in which dysregulated CD86 expression on tumor-infiltrating APCs could critically influence the balance between anti-tumor immunity and immune evasion.

Trans-endocytosis (TE) represents a critical regulatory mechanism whereby CD152 captures CD80 or CD86 from opposing APCs, leading to ligand internalization and lysosomal degradation [26,42]. Importantly, the intracellular fate of each ligand differs. CD80 remains stably bound to CD152 and is subsequently ubiquitylated and targeted to late endosomes and lysosomes for degradation. In contrast, CD86 dissociates from CD152 within the acidic endosomal compartment in a pH-dependent manner, permitting CD152 recycling to the cell surface and repeated rounds of TE. Disruption of CD86–CD152 trans-endocytosis has been associated with the development of autoimmunity, underscoring the physiological importance of this pathway [26,42,43].

Upon exposure to inflammatory stimuli such as LPS or antigenic challenge, downregulation of MARCH1 allows newly synthesized CD86 to escape degradation and accumulate at high density on the DC membrane [41]. This surge in CD86 effectively overrides pre-existing suppressive CD80–CD152 signaling and provides a strong second signal for T-cell activation. In synergy with stimulatory cytokines, particularly IL-12p70, mature DCs drive robust T-cell activation, clonal expansion, and Th1 differentiation, characterized by high IFN-γ secretion and cytotoxic responses [19]. As T cells proliferate, CD152 is upregulated; however, because CD86 has lower affinity for CD152, it is less efficiently sequestered, allowing sustained effects on T-cell division and maintenance of the immune response [36,42,44,45].

Kennedy et al. demonstrated that CTLA-4 preferentially regulates immune responses by repeatedly removing CD86 from APCs, thereby establishing CD86 as the principal ligand controlling CD28-mediated co-stimulation and immune tolerance [42]. Although CD80 and CD86 share the same receptors and function as co-stimulatory ligands together with MHC-mediated antigen presentation, their differential expression kinetics, binding affinities, and structural properties suggest complementary rather than redundant roles in regulating T-cell activation and immune tolerance [12,18,42]. In particular, its rapid induction, distinct receptor affinity, and dynamic intracellular trafficking position CD86 as a critical and context-dependent determinant of co-stimulatory signaling during T-cell priming and anti-tumor immunity [18,42,43].

In addition to membrane-bound CD86, soluble CD86 (sCD86) has been detected in circulation and may exert immunomodulatory functions [46,47,48]. sCD86 can interact with CD28 or CTLA-4, potentially influencing T-cell activation or immune tolerance in a context-dependent manner [46,49,50]. Although its precise biological role remains incompletely understood, emerging evidence suggests that sCD86 has been investigated in association with immune activation and dysregulated immune states in cancer [47,48,51].

The molecular origin of sCD86 remains under investigation, with proposed mechanisms including alternative mRNA splicing and proteolytic cleavage of membrane-bound CD86 [47,52]. Functionally, sCD86 may act as a competitive inhibitor or decoy receptor, potentially modulating co-stimulatory signaling under specific conditions [53]. However, its biological and clinical significance requires further validation.

Both membrane-bound and soluble CD86 contribute to the complexity of CD86-mediated immune regulation. Rather than functioning as a static maturation marker, CD86 appears to be continuously shaped by MARCH1-mediated ubiquitination, CTLA-4-driven trans-endocytosis, soluble ligand biology, and tumor microenvironmental stress.

**Figure 1:**
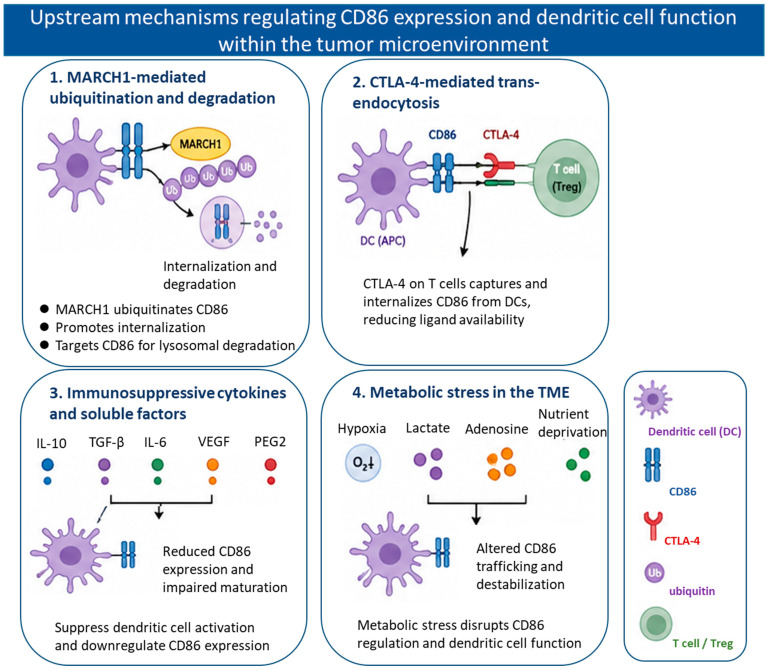
Upstream mechanisms regulating CD86 expression and dendritic cell function within the tumor microenvironment.

These findings indicate that CD86 regulation is influenced by co-stimulatory signaling, post-translational regulation, and tumor microenvironment-associated immune modulation (Figure 1).

(1) Under steady-state conditions, MARCH1-mediated ubiquitination promotes CD86 internalization and lysosomal degradation, thereby maintaining low surface CD86 expression.
(2) CTLA-4 expressed on activated T cells or regulatory T cells removes CD86 from antigen-presenting cells through trans-endocytosis, reducing ligand availability and attenuating co-stimulatory signaling.
(3) Immunosuppressive cytokines and soluble factors, including IL-10, TGF-β, IL-6, VEGF, and PGE2, impair dendritic cell maturation and suppress CD86-associated co-stimulatory function.
(4) Tumor-associated metabolic stress, including hypoxia, lactate accumulation, adenosine, and nutrient deprivation, alters dendritic cell function and may disrupt CD86 surface stability and trafficking.

These integrated upstream regulatory mechanisms dynamically shape CD86 expression and contribute to context-dependent dendritic cell function in cancer immunity.

Abbreviations: APC, antigen-presenting cell; CD86, cluster of differentiation 86; CTLA-4, cytotoxic T-lymphocyte-associated protein 4; DC, dendritic cell; HIF-1α, hypoxia-inducible factor 1 alpha; IL, interleukin; LPS, lipopolysaccharide; MARCH1, membrane-associated RING-CH-type finger 1; PGE2, prostaglandin E2; sCD86, soluble CD86; TGF-β, transforming growth factor beta; TME, tumor microenvironment; Treg, regulatory T cell; VEGF, vascular endothelial growth factor.

## 3. Regulation of CD86 Expression in Cancer-Associated Myeloid Cells

Myeloid cells constitute a highly versatile and heterogeneous lineage within the innate immune system. These cells play a prominent role in cancer pathophysiology and exhibit remarkable plasticity, ranging from anti-tumor to pro-tumor phenotypes depending on signals within the TME [54]. This population includes tumor-associated macrophages (TAMs), tumor-associated neutrophils (TANs), myeloid-derived suppressor cells (MDSCs), dendritic cells, and eosinophils [55,56,57]. These populations display considerable functional plasticity within the tumor microenvironment and may contribute to either immune activation or immune suppression depending on local signaling conditions [58,59].

The expression of CD86 is tightly modulated by immune signaling cascades and by the chronic inflammatory milieu of the TME, both of which ultimately affect DC function. Whereas acute inflammation generally upregulates CD86 expression, the chronic smoldering inflammation of the TME establishes negative feedback loops that suppress CD86 [60]. The persistent presence of inflammatory mediators, hypoxia, nutrient deprivation, acidity, and dysregulated production of growth factors—including granulocyte-macrophage colony-stimulating factor (GM-CSF), granulocyte colony-stimulating factor (G-CSF), and macrophage colony-stimulating factor 1 (CSF1)—promotes the generation, expansion, and accumulation of MDSCs [61,62,63,64]. Once established, this chronic inflammatory milieu can shift the immunologic balance away from effective T-cell priming [64].

Within suppressive networks involving MDSCs, TAMs, and Tregs, CD86 expression is actively inhibited by immunosuppressive cytokines, most notably IL-6, IL-10, transforming growth factor-β (TGF-β), prostaglandin E2 (PGE2), and vascular endothelial growth factor (VEGF) [62,65,66]. These factors hinder DC maturation, downregulate MHC and co-stimulatory molecule expression, and limit production of IL-12, a cytokine essential for potent anti-tumor responses [67,68,69]. Specifically, MDSC-derived VEGF and IL-10 activate signal transducer and activator of transcription 3 (STAT3), subsequently downregulating MHC class II and co-stimulatory molecules such as CD86 on DCs [63]. IL-6 derived from immune cells, tumor cells, endothelial cells, and fibroblasts also utilizes the STAT3 pathway to reduce MHC-II, IL-12p70, and CD86 expression [70]. Presentation of tumor-associated antigens in the absence of these essential co-stimulatory signals leads to T-cell anergy [68,71,72]. Furthermore, MDSC-derived VEGF impairs DC development by inhibiting the activity of FMS-related tyrosine kinase 3 ligand (FLT3L), which is primarily produced by NK cells and is indispensable for DC proliferation and survival [3,14]. Under these suppressive conditions, tumor-conditioned DCs produce TGF-β1, which further attenuates CD86 expression and promotes induction of CD4+CD25+Foxp3+ Tregs [66,73].

As discussed above, CD86 is considered to play an important role in T-cell priming and may influence anti-tumor immune responses. In solid tumors, CD86 is often suppressed, and higher expression has generally been associated with more favorable immune activity. Nevertheless, CD86 may be upregulated in selected contexts and could potentially contribute to tumor persistence by promoting Treg expansion [74,75].

In summary, cancer-associated myeloid cells frequently suppress dendritic cell maturation and co-stimulatory capacity, thereby representing a major barrier to effective DC-based immunotherapy. Within this regulatory network, CD86 expression should be interpreted together with broader changes in antigen presentation, cytokine production, and local immune suppression rather than as an isolated maturation marker.

Metabolic stress within the TME may further impair dendritic cell maturation. Hypoxia-driven stabilization of hypoxia-inducible factor-1α (HIF-1α) and the accumulation of metabolites such as lactate have been associated with tolerogenic myeloid phenotypes and weakened T-cell priming [76,77,78]. Although these conditions may influence CD86 expression indirectly, direct causal links between specific metabolic pathways and CD86 surface regulation remain incompletely defined and require further mechanistic validation.

Importantly, CD86-mediated co-stimulation is initiated primarily during dendritic cell-T-cell interactions in secondary lymphoid organs and may subsequently influence downstream anti-tumor immune responses within tumor tissues [79]. Therefore, CD86 regulation should be interpreted across spatial compartments and temporal stages of the immune response. Major regulatory mechanisms influencing CD86 expression and dendritic cell function within the tumor microenvironment are summarized in Table 1.

## 4. Regulation of CD86 Across Immune Contexts

### 4.1. Regulatory Complexity of CD86

CD86 expression is regulated by inflammatory signaling, immune checkpoint interactions, post-translational modification, and metabolic stress within the tumor microenvironment (TME). Rather than serving solely as a static maturation marker, CD86 surface expression may vary according to dendritic cell subset composition, inflammatory conditions, and local immune regulation.

Conventional dendritic cell type 1 (cDC1) subsets are specialized for antigen cross-presentation and CD8+ T-cell activation, whereas cDC2 subsets primarily support CD4+ T-cell priming and helper T-cell differentiation [31,32]. Plasmacytoid dendritic cells (pDCs) may acquire tolerogenic features under chronic inflammatory conditions [33,34], while monocyte-derived dendritic cells (moDCs) generated during inflammatory responses may exhibit distinct co-stimulatory profiles depending on cytokine and metabolic signaling [35]. These findings suggest that CD86 regulation may differ across dendritic cell lineages and immune conditions.

Under inflammatory stimulation, transient CD86 upregulation supports T-cell priming and effector immune activation [19,36,44,45]. In contrast, chronic exposure to suppressive cytokines such as IL-10, TGF-β, and VEGF within the TME may impair dendritic cell maturation and attenuate co-stimulatory signaling through STAT3-associated pathways and immunosuppressive signaling [62,63,65,66,67,68,69,70]. Hypoxia and lactate accumulation may further contribute to tolerogenic immune phenotypes and impaired T-cell priming [76,77,78].

Post-translational regulation also contributes to CD86 surface stability. MARCH1-mediated ubiquitination promotes CD86 internalization and degradation under steady-state conditions, whereas CTLA-4-mediated trans-endocytosis regulates CD86 availability during dendritic cell–T-cell interactions [26,40,41,42,43].

Importantly, CD86-mediated co-stimulation occurs both in secondary lymphoid organs and within tumor-associated immune niches [79,80]. Therefore, interpretation of CD86 expression should consider dendritic cell subset composition, immune microenvironment, and spatial immune context.

Current evidence supports that CD86 regulation is influenced by integrated immune and microenvironmental signals within the TME [26,40,41,42,43,62,63,65,66,67,68,69,70,76,77,78]. However, the relative contribution of individual regulatory pathways in different cancer settings remains incompletely understood. This integrated regulatory complexity also complicates the interpretation of experimental findings across different model systems.

Importantly, most mechanistic insights regarding CD86 regulation have been derived from murine bone marrow-derived dendritic cell models [26,40,41,42,43]. However, human dendritic cells display greater phenotypic diversity [3,14,31,32], tissue-specific adaptation, and functional plasticity. These interspecies differences may substantially affect CD86 expression dynamics and immune consequences, highlighting an important translational gap between experimental and clinical observations. These differences may partly explain why CD86-associated immune outcomes observed in preclinical murine models are not consistently reproduced in human tumor studies.

### 4.2. Current Limitations and Future Directions

Current evidence supports a role for CD86 in dendritic cell-mediated immune regulation; however, several important questions remain unresolved.

First, the mechanisms governing temporal and spatial regulation of CD86 across different tumor microenvironments require further clarification. Future studies incorporating longitudinal analyses, single-cell and spatial transcriptomic approaches, and functional immune profiling may help clarify how CD86 expression dynamically changes during tumor progression and in response to immunotherapy [31,54,62].

Second, additional mechanistic studies are needed to clarify how metabolic stress, suppressive cytokines, and immune checkpoint pathways coordinately regulate CD86 surface stability and co-stimulatory signaling. In particular, the interaction between CD86 and CTLA-4 represents an important regulatory axis in dendritic cell–T-cell communication and immune tolerance [26,42,43].

Third, the clinical relevance of dynamic CD86 regulation remains to be established. Although CD86 has been investigated in relation to immune activation and dendritic cell functional status, its clinical relevance remains incompletely understood and requires further validation across different cancer types. Standardized detection methods and functional validation in independent patient cohorts will be necessary before potential clinical application.

A more comprehensive understanding of CD86 regulation may provide further insight into dendritic cell biology and tumor-associated immune regulation. Future studies integrating mechanistic investigation with functional immune analyses may provide a stronger basis for evaluating the functional relevance of CD86 regulation in cancer immunity.

### 4.3. Controversies and Unresolved Questions

Despite extensive investigation, several controversies regarding CD86 remain unresolved. First, whether CD86 primarily functions as a maturation marker or as an active immunoregulatory molecule remains debated [18,19,42]. Second, elevated CD86 expression does not universally indicate favorable anti-tumor immunity, as it may also associate with regulatory T-cell expansion and immune tolerance under suppressive tumor contexts [36]. Third, the functional role of soluble CD86 remains controversial, with reports suggesting both co-stimulatory and immunosuppressive activities [46,47]. Finally, whether the intensity of CD86 expression reflects dendritic cell activation, immune exhaustion, or compensatory regulation remains poorly understood [62,74,75]. Resolving these questions will be essential for defining the biological and clinical significance of CD86 in cancer immunity.

## 5. Clinical Implications and Translational Perspectives

**Figure 2:**
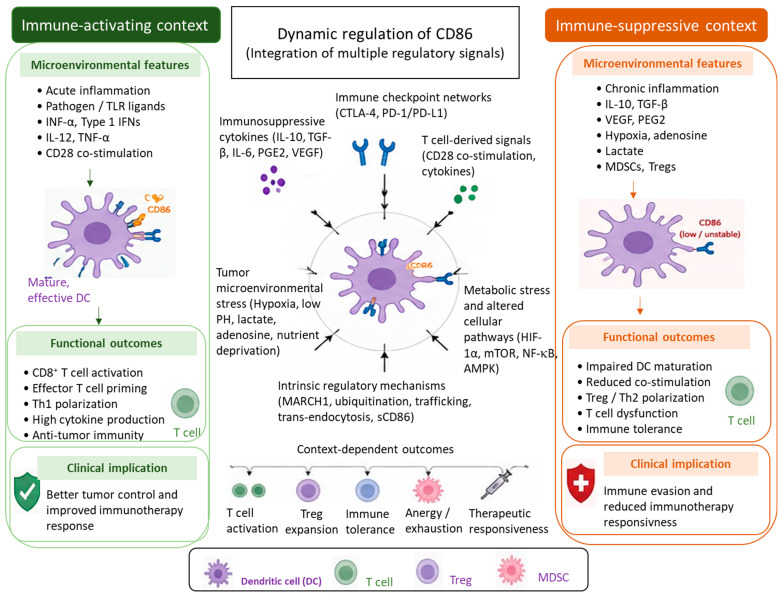
Integrated conceptual model of context-dependent CD86 function in cancer immunity. Extrinsic regulatory factors, including CTLA-4-mediated trans-endocytosis, immunosuppressive cytokines (IL-10, TGF-β, and VEGF), and tumor-associated metabolic stress (hypoxia, lactate accumulation, and adenosine), dynamically influence CD86 surface stability, signaling capacity, and functional availability on dendritic cells.

The context-dependent regulatory and functional implications of CD86 in cancer immunity are summarized in Figure 2.

Under immune-activating conditions, preserved CD86 expression supports effective T-cell priming, cytotoxic immune activation, and anti-tumor immunity. In contrast, under immunosuppressive tumor microenvironments, altered CD86 regulation may favor regulatory T-cell expansion, immune tolerance, dysfunctional T-cell responses, and tumor immune escape.

This model highlights the dual and context-dependent role of CD86 as both a co-stimulatory molecule and a dynamic immunoregulatory molecule, with potential implications for prognosis, therapeutic responsiveness, and cancer immunotherapy.

### 5.1. The Context-Dependent Role of CD86 in Cancer Prognosis

The prognostic significance of CD86 expression is highly context-dependent, reflecting its dual immunological roles across different malignancies [36,74,81,82,83]. Pan-cancer analyses from The Cancer Genome Atlas (TCGA) have shown that elevated CD86 levels correlate with improved overall survival in cervical carcinoma and cutaneous melanoma, where higher CD86 expression has been reported in association with favorable immune activity and increased immune infiltration in selected studies [81,82]. Conversely, in lower-grade glioma, glioblastoma, and acute myeloid leukemia, high CD86 expression has been linked to inferior outcomes and increased resistance to therapy [81,83].

These findings suggest that evaluating CD86 solely as a static maturation marker is insufficient. Instead, assessment of temporal and context-dependent changes in CD86 expression may provide additional insight into dendritic cell functional states and tumor-associated immune regulation. CD86-expressing dendritic cells within tertiary lymphoid structures (TLSs) may participate in local T-cell priming and have been reported in association with favorable immune contexture and increased immune infiltration in selected studies [80]. Importantly, CD86 expression has been investigated in relation to immune activation and dendritic cell functional status; however, its clinical relevance remains incompletely understood and requires further validation across different cancer types. Standardization of detection methods and validation across independent cohorts will be essential for future clinical translation.

Mechanistically, elevated CD86 expression may not always indicate effective anti-tumor immunity. In certain suppressive tumor microenvironments, persistent CD86 expression may support CTLA-4 engagement and preferential Treg maintenance rather than productive effector T-cell activation [36,42,43]. In Treg-enriched tumors, high CD86 may preferentially reinforce inhibitory immune circuits rather than productive anti-tumor immunity. This duality may partly explain why CD86 is associated with a favorable prognosis in immune-inflamed tumors but poor outcomes in Treg-enriched or chronically suppressed tumor settings.

### 5.2. Potential Translational Implications of CD86 Regulation

CD86 contributes to the dynamic balance between immune activation and immune tolerance through its interactions with co-stimulatory and co-inhibitory pathways. Its functional significance depends on dendritic cell state, immune checkpoint interactions, and suppressive signals within the tumor microenvironment.

Beyond its mechanistic role, dynamic monitoring of CD86 may serve as an immunological indicator rather than a standalone predictive biomarker for immunotherapy responsiveness. Since effective immune checkpoint blockade partially depends on intact co-stimulatory signaling, temporal changes in CD86 expression may reflect immune competence and treatment sensitivity [84,85]. This possibility warrants prospective evaluation in clinical immunotherapy cohorts.

From a clinical perspective, CD86 has been explored as a potential indicator of immune activation and dendritic cell functional status, particularly in the context of dendritic cell activation and T-cell priming. However, its clinical utility remains to be validated in large, well-characterized patient cohorts. In addition, the relative contributions of membrane-bound CD86 and soluble CD86 (sCD86) to immune regulation are not yet fully understood, and further investigation is required to clarify their respective roles in cancer progression and therapeutic response.

From a translational perspective, modulation of dendritic cell activation and co-stimulatory signaling may represent a potential strategy to improve anti-tumor immune responses. However, the clinical relevance of dynamic CD86 regulation remains incompletely understood and requires further validation in mechanistic and translational studies.

Importantly, CD86-related pathways may represent potential targets for immunomodulatory strategies. While these findings suggest potential therapeutic relevance, clinical evidence supporting CD86-targeted strategies remains limited. Given its interaction with CD28 and CTLA-4, modulation of CD86 signaling could influence the efficacy of immune checkpoint blockade therapies [42,43,49]. Notably, effective PD-1/PD-L1 blockade has been shown to depend on CD28-mediated co-stimulation [84,85], suggesting that CD86-regulated T-cell priming may indirectly shape therapeutic responses [14,85]. However, direct clinical evidence linking CD86 modulation to checkpoint inhibitor efficacy remains limited. In this context, CD86 may not function as a standalone therapeutic target but rather as part of a broader regulatory network that shapes anti-tumor immunity. Therefore, strategies integrating CD86 modulation with existing immunotherapies may warrant further exploration. Combination strategies integrating dendritic cell activation, immune checkpoint blockade, and innate immune agonists may further modulate CD86-associated immune regulation.

Despite these advances, several critical knowledge gaps remain. First, the mechanisms governing context-dependent regulation of CD86 across different tumor microenvironments are incompletely understood. Second, the functional heterogeneity of CD86 expression among dendritic cell subsets and its impact on downstream immune responses require further clarification. Third, the clinical relevance of CD86 dynamics, including its temporal and spatial regulation, has yet to be systematically evaluated.

Current evidence suggests that CD86 functions as a context-dependent regulator of immune balance within the tumor microenvironment. The major context-dependent functional implications of CD86 regulation in cancer immunity are summarized in Table 2.

These observations underscore the need to evaluate CD86 regulation using mechanistic and functional approaches rather than relying solely on static expression measurements.

## 6. Future Perspectives

Future studies should define how CD86 expression and surface stability are regulated across dendritic cell subsets, anatomical compartments, and stages of tumor progression. Longitudinal immune profiling, single-cell analyses, and paired functional assays may help clarify whether CD86 changes reflect dendritic cell activation, immune suppression, or altered cellular composition within the TME. Emerging single-cell and spatial transcriptomic technologies may help resolve the spatial and functional heterogeneity of CD86-expressing dendritic cell populations within distinct tumor microenvironments [86].

Mechanistic studies incorporating T-cell co-culture systems, IFN-γ secretion profiling, cytotoxicity assays, and perturbation of upstream regulators such as IL-10/STAT3, TGF-β, hypoxia-related signaling, and CTLA-4-mediated trans-endocytosis will be important for determining the functional impact of CD86 regulation in cancer immunity [87,88].

Integration of spatial immunology, single-cell transcriptomics, and functional immune profiling may further refine the understanding of CD86-associated immune regulation in human cancers.

## 7. Conclusions

Available evidence supports that CD86 participates in dendritic cell-mediated immune regulation through interactions involving co-stimulatory signaling, immune checkpoint pathways, and tumor microenvironment-associated immune modulation. CD86 expression may vary across inflammatory conditions, dendritic cell subsets, and spatial immune contexts within the tumor microenvironment.

Although accumulating studies have investigated CD86 in relation to dendritic cell activation and anti-tumor immunity, the mechanistic and clinical significance of CD86 regulation remains incompletely understood. Further mechanistic, spatial, and translational studies will be necessary to clarify the functional relevance of CD86 regulation across different cancer settings.

## Figures and Tables

**Table 1:** Major mechanisms regulating CD86 expression and function in the tumor microenvironment.

Regulatory Factor	Mechanism	Effect on CD86/DC Function	References
MARCH1	Ubiquitination	CD86 internalization/degradation	[40,41]
CTLA-4	Trans-endocytosis	removal of CD86 from APCs	[42,43]
IL-10	STAT3 activation	suppression of DC maturation	[62,68,69]
TGF-β	Immunosuppressive signaling	reduced co-stimulation	[65,66,67]
VEGF	Impaired DC differentiation	decreased CD86 expression	[62,63]
Hypoxia	HIF-1α-mediated stress	tolerogenic DC phenotype	[76,77]
Lactate	Metabolic dysregulation	impaired T-cell priming	[78]

**Table 2:** Reported associations of CD86 regulation in different immune contexts.

Context	CD86-Associated Features	Potential Immune Consequence	Reported Association
Acute inflammatory activation	Transient CD86 upregulation; enhanced co-stimulatory signaling	Efficient T-cell priming and Th1 activation	Association with enhanced anti-tumor immune activity
Chronic suppressive TME	Reduced CD86 expression and impaired DC maturation	Weak T-cell activation and immune tolerance	Association with immune suppression
CTLA-4-mediated regulation	Trans-endocytosis of CD86	Reduced co-stimulatory signaling	Immune checkpoint–associated suppression
Metabolic stress/hypoxia	Altered DC function and tolerogenic phenotype	Impaired anti-tumor immune responses	Context-dependent immunosuppression
TLS-associated immune niches	Localized CD86-expressing dendritic cell populations	Enhanced local T-cell priming	Reported association with favorable immune contexture
DC subset heterogeneity	Differential CD86 regulation among cDC1/cDC2/pDC/moDC	Variable immune activation capacity	Functional heterogeneity across TMEs
Spatial immune context	Spatially heterogeneous CD86 regulation across lymphoid structures and tumor immune niches	Regional variability in T-cell priming and immune activation	Reported association with spatial immune heterogeneity

## Data Availability

No new data were created or analyzed in this study. Data sharing is not applicable to this article.

## Abbreviations

The following abbreviations are used in this manuscript:

DC	Dendritic cell
DCs	Dendritic cells
TME	Tumor microenvironment
MHC	Major histocompatibility complex
CTLA-4	Cytotoxic T-lymphocyte-associated protein 4
APCs	Antigen-presenting cells
LPS	Lipopolysaccharide
TLR	Toll-like receptor
TLS	Tertiary lymphoid structures
Treg	Regulatory T cell
Th1	T helper 1
NK	Natural killer
TE	Trans-endocytosis
TAMs	Tumor-associated macrophages
TANs	Tumor-associated neutrophils
MDSCs	Myeloid-derived suppressor cells
GM-CSF	Granulocyte–macrophage colony-stimulating factor
G-CSF	Granulocyte colony-stimulating factor
CSF-1	Colony-stimulating factor 1
TGF-β	Transforming growth factor-β
PGE2	Prostaglandin E2
VEGF	Vascular endothelial growth factor
STAT3	Signal transducer and activator of transcription 3
FLT3L	FMS-related tyrosine kinase 3 ligand
ICIs	Immune checkpoint inhibitors
TCGA	The Cancer Genome Atlas
